# Watershed segmentation of basal left ventricle for quantitation of cine cardiac MRI function

**DOI:** 10.1186/1532-429X-13-S1-P4

**Published:** 2011-02-02

**Authors:** Yingli Lu, Kim A Connelly, Alexander J Dick, Graham A Wright, Perry E Radau

**Affiliations:** 1 Imaging Research, Sunnybrook Health Sciences Centre, Toronto, ON, Canada; 2 Cardiology, St Michael's Hospital, Toronto, ON, Canada; 3 Cardiology, Sunnybrook Health Sciences Centre, Toronto, ON, Canada

## Introduction

To quantitatively analyze global and regional cardiac function from MR, clinical parameters such as ejection fraction (EF) and volumes are required. These depend upon accurate delineation of endo- and epicardial contours of the left ventricle (LV). Previous work [1] has demonstrated the difficulty of accurate LV segmentation, especially in basal slices where the LV outflow tract (LVOT) interrupts continuous myocardial contours. A novel method for the robust, accurate and fully automatic LV segmentation from short axis (SA) cine MR images is presented in this study that applies watershed technique to solve basal slice segmentation.

## Materials and methods

**Figure 1:**
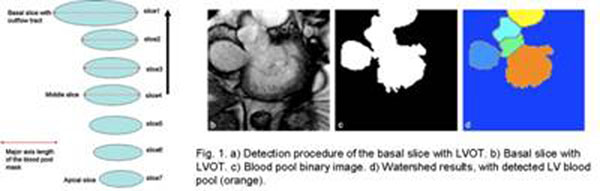
a) Detection procedure of the basal slice with LVOT. b) Basal slice with LVOT. c) Blood pool binary image. d) Watershed results, with detected LV blood pool (orange).

Imaging data (N=146, 40 ischemic heart failure, 32 non-ischemic heart failure, 35 LV hypertrophy and 39 normals; 37 female, 109 male; age: 59.815.8) were acquired from a 1.5T scanner (GE CV/i Excite) with IR-SSFP SA cine MR. The basal slice with LVOT is identified by the following steps (Fig. 1a): 1. Choose the middle slice image as the start image, and process each image sequentially in the basal direction. 2. Apply the optimal threshold method [1] to convert the ROI to a binary image (Fig.1b). 3. Identify the binary object with blood pool and LVOT (Fig.1b). 4. Calculate the length of the major axis ***L*** of the ellipse that has the same normalized second central moments as the binary object. 5. If the ratio of current ***L*** to preceding ***L*** larger than a predefined threshold (In this work, threshold = 1.2), basal slice with LVOT is identified.

The blood pool is separated from the LVOT by the following steps: 1. Calculate the Euclidean distance transform of the binary object, i.e., the distance between the pixel and it’s nearest zero pixel of the binary object. 2. Compute the watershed regions of the distance image (Fig. 1c).

## Results

The average perpendicular distance (APD) between the detected and the manually drawn expert contours and Dice metric (DM) over slices and ES and ED phases are shown in Table 1. The average computation time of the watershed segmentation is about 0.027s per image. The reproducibility of this automated technique is 100%.

**Table 1:** Evaluation of contours

Patient Group	LV located (%)	APD (mm)		DM	
		endo	epi	endo	epi
HFI	97.5 (39/40)	1.83	1.86	0.92	0.94
HFNI	96.9 (31/32)	1.91	1.93	0.92	0.94
HYP	94.3 (33/35)	2.70	2.14	0.85	0.93
HEA	89.7 (35/39)	1.88	1.87	0.90	0.93

endo: endocardial, epi: epicardial, APD: average perpendicular distance, DM: Dice metric, HFI: heart failure with ischemia, HNFI: heart failure, no ischemia, HYP: hypertrophy, HEA: healthy, LV: left ventricle.

## Discussion and conclusions

The proposed fully automated segmentation technique, with an application of watershed algorithm to basal slices, is fast, accurate and reproducible. It should be of benefit for quantification of cine cardiac MR in clinical practice.
